# Multiplicity in public health supply systems: a learning agenda

**DOI:** 10.9745/GHSP-D-12-00042

**Published:** 2013-06-26

**Authors:** Alan Bornbusch, James Bates

**Affiliations:** a U.S. Agency for International Development, Bureau for Global Health, Washington, DC, USA; b John Snow, Inc., Arlington, VA, USA

## Abstract

Supply chain integration—merging products for health programs into a single supply chain—tends to be the dominant model in health sector reform. However, multiplicity in a supply system may be justified as a risk management strategy that can better ensure product availability, advance specific health program objectives, and increase efficiency.

Conventional wisdom in health sector reform tends to favor **supply chain integration**—merging supply chain functionalities, such as distribution, across different health programs—to improve efficiency and health systems overall. Supply chain research and application in the commercial sector, however, point toward **multiplicity in supply systems**—that is, structuring a supply system to take advantage of multiple supply chains or supply chain segments to reduce risk and maintain supply.

We explore the role that multiplicity has played historically in public health supply systems and consider recent examples where multiplicity has been introduced to reduce risk and improve system performance. The limited, but suggestive, evidence from public health supply systems thus far, combined with recent methodological advances, point to the need and opportunity for further inquiry into the case for multiplicity in public health supply systems in low- and middle-income countries.

## THE CENTRAL MEDICAL STORE MODEL

In Africa, Asia, and Latin America, the **“central medical store” (CMS) supply chain model** dominates in public-sector health programs—a model that is both administratively and physically centralized. Procurement typically takes place at the national level; most stock enters distribution through a centrally located warehouse in the capital city; and a public-sector entity implements (or at least oversees) distribution.

CMSs have long been perceived to be burdened with the inefficiencies and shortcomings widely associated with monopolistic systems, and sometimes are even described as “monopolies.” The term is not inappropriate. In most cases, CMSs have handled a predominate share of supplies distributed within the public sector. More importantly, their roles have often been protected by law, and they usually have not been threatened with losing their business to more efficient systems.^1^-^5^

## THE ALTERNATIVE MODEL OF MULTIPLICITY

Rarely, however, are CMSs true monopolies, as they almost always operate in the company of other supply chains. A hybrid state often prevails, more appropriately characterized as a supply network or system.

Indeed, ministries and donors have introduced alternative supply chains or supply chain segments since the earliest days of development assistance, including:

- Donor-managed procurement and distribution to countries
- Nongovernmental organizations (NGOs) taking on procurement and distribution responsibilities
- “Vertical” supply chains serving the distribution needs of specific health programs
- Country governments purchasing services from private logistics providers on a routine basis

Considering this history, **3 principal motives** emerge for moving outside routine CMS operations and toward multiplicity:

1. Situation-specific problem solving
2. Advancement of priority health program objectives
3. Pursuit of efficiency through options-building

We explore these motives further in the **3 mini-case studies** that follow. All 3 motives represent forms of risk management,* whether it be managing the risk of non-availability of essential products; inability to provide priority health services; or higher than necessary acquisition and operating costs.

Many of these risks and the approaches taken to manage them resemble those studied in supply chain research and applied in commercial sector practice. Indeed, multiplicity, including managing multiple supplier pipelines or segmenting supply chains, is an established risk management approach in commercial supply systems.^7^-^10^ (Supply chain segmentation is one way to organize a supply system into specialized supply chains or supply chain segments that are tailored to groups of products that share certain characteristics, such as demand patterns.^11^-^13^)

3 key advantages of multiplicity in supply chains: ensuring product availability, advancing priority health objectives, and greater efficiency.

### Uganda: Situational Problem Solving

Situation-specific problem solving in supply chain management can be thought of as activities (for example, procurement, storage, and transport) that are deployed in an ad hoc manner to respond to events, such as disease outbreaks, natural disasters, or management of a donor-required competitive bidding process. Ad hoc solutions are also sometimes needed to address more general supply chain breakdowns that threaten supply shortages. The solution to an immediate problem can prove valuable in more than just the short term, revealing opportunities for overall improvement in a supply system. A case in point comes from Uganda.

In early 2010, demand for family planning supplies managed by the public sector's National Medical Stores (NMSs) rapidly increased. Simultaneously, policy changes and a significant gap in funding for commodities made it more difficult for NGOs and faith-based organizations (FBOs) to obtain supplies from the public sector. This led to widespread contraceptive stockouts at private-sector community-based facilities.

In response, a new supply chain was created, managed by a local social marketing organization. Members of the Uganda Family Planning Coalition, formed by Uganda's leading family planning providers in response to the crisis, were the first to receive supplies through this channel. The channel has since grown into a supply chain used by the government and donors to deliver reproductive health commodities to NGOs, FBOs, and small for-profit providers (with an expected value of US$20 million for 2013–2014). The creation of a supply chain to serve private-sector providers alongside the NMS-managed system marks an important step forward in increasing access to contraceptives in Uganda (personal communication with Linda Cahaelen, Senior Technical Advisor, USAID, 2012).

A contraceptive supply chain in Uganda, created initially to solve short-term stockout problems at private facilities, now delivers reproductive health commodities valued at US$20 million.

### Bangladesh: Advancing Priority Program Objectives

In some places, donor and country partners set up program-specific supply chains that operate alongside the CMS system to advance certain priority health program objectives. Family planning, immunization, malaria, and tuberculosis are among the best known. These so-called vertical supply chains are often judged in health reform circles as inefficient and counterproductive in strengthening a more integrated CMS. In most cases, their good track record suggests a more complicated picture.

Supply chain investments for contraceptives have contributed to the success of the Bangladesh family planning program.

Although concern about financial sustainability is valid, critics of vertical supply chains tend to overlook the health program growth and public health impacts of these systems. Consider Bangladesh, where use of modern contraception has grown nearly 10-fold from 1975 to the present. Investments in supply chains dedicated largely to family planning products in the public and private (social marketing) sectors are widely credited to the phenomenal success of family planning programs in Bangladesh.^14^-^15^ Indeed, several multiplicities have operated:

- Within the public sector, two separate supply chains, with similar functional capabilities but applied to different products, operate—one mostly for family planning products and the second for other essential medicines.
- The public sector and social marketing supply chains for family planning have at times functioned as if parts of a coordinated system, exchanging products when one or the other experiences shortages.
- The public sector has employed multiple distribution options, contracting with commercial carriers to cover parts of the country.

By ensuring a reliable supply of contraceptive products, these supply chains have helped to engender a public expectation of and demand for widespread family planning services—an important ingredient to the long-term sustainability of family planning in Bangladesh.

### Chile: Building Options

Between 1970 and 2010, the CMS in Chile known as CENABAST (Central de Abastecimiento) provided procurement and distribution services for all essential health commodities to 26 regional health authorities.^16^ In the early 2000s, Chile decentralized its health management system. The government gave regional health offices latitude to purchase through CENABAST or from local distributors. Overall, CENABAST did not suffer from dysfunction during this period, and most regional health offices continued to use CENABAST despite having other options.^17^

In addition to using CENABAST, in 2010 regional health offices also began purchasing commodities through ChileCompra (“Chile Buys”), a government-wide e-procurement service.^18^ ChileCompra negotiates multi-year agreements with suppliers for products that, for example, have high volume and predictable demand. Using an electronic catalogue, government agencies can take advantage of the lower prices negotiated by ChileCompra and avoid the expense and delays of issuing individual tenders.^19^

In Chile, the Ministry of Health had not set out to purposely build multiplicity (see Figure). Rather, other developments, such as decentralization, procurement reform, and segmentation of procurement between CENABAST and ChileCompra for greater efficiency, have promoted this result.

**Figure.**
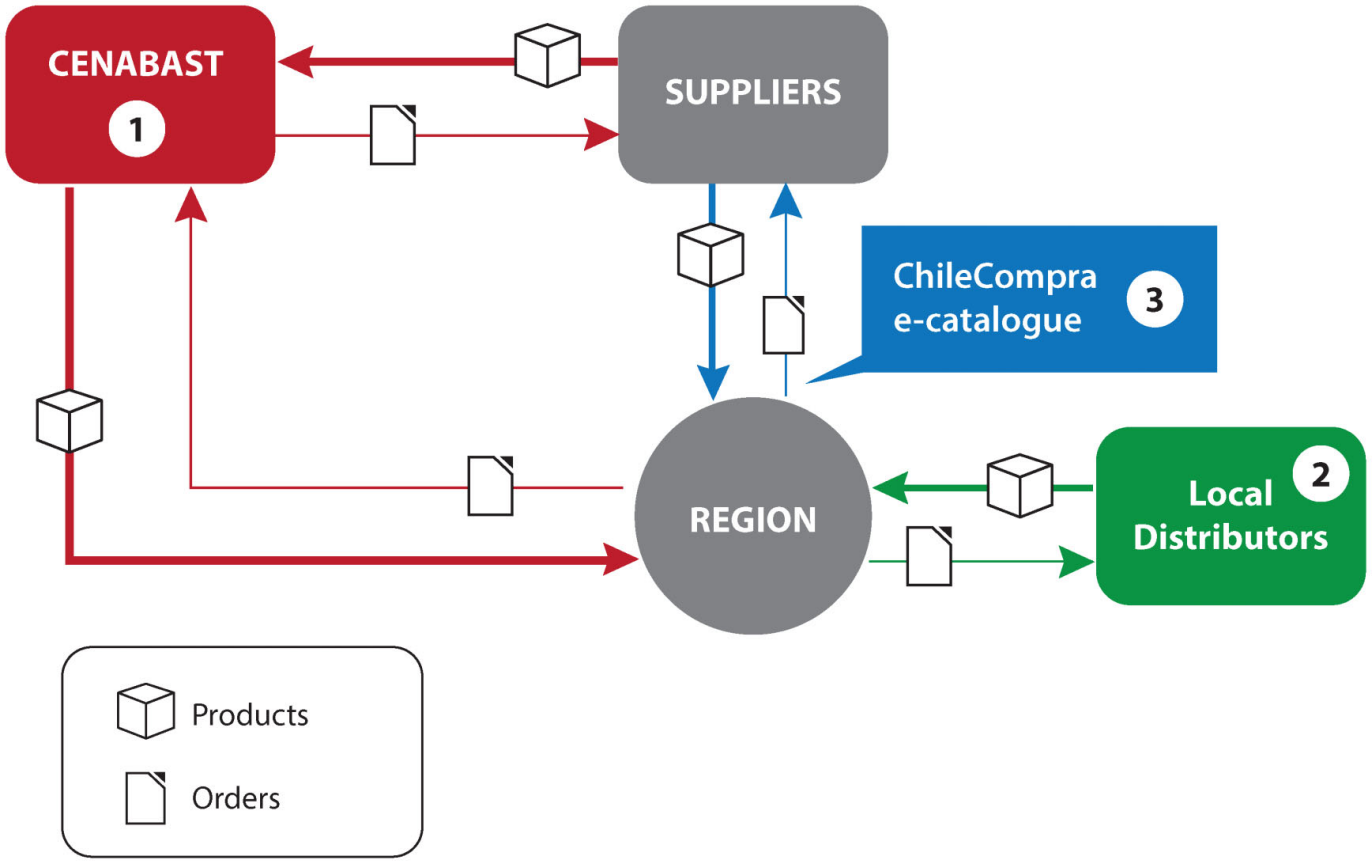
A Supply System With Multiplicity Regional health authorities in Chile have several options to procure health commodities: (1) from CENABAST (the central medical store), who in turn procures from suppliers (red lines); (2) from local distributors (green); or (3) directly from suppliers through the ChileCompra e-catalogue, using pre-established agreements (blue). The Ministry of Health has overall oversight for the system.

## A LEARNING AGENDA

Is there a downside to multiplicity in supply systems? There can be. For example, multiplicity has in all likelihood been carried to excess where there is an over-proliferation of program- or donor-specific supply chains.^20^-^22^

**Advantages** of multiplicity include:

- Greater flexibility to maintain supply
- Competition can lower costs and improve service levels
- Supply chains or segments can be tailored to program- or product-specific needs

**Disadvantages** include:

- Need for increased oversight, management, and coordination across the supply system
- Increased cost and potential for inefficiency
- Over-specialization can reduce flexibility

Although these pros and cons are to a certain degree intuitive, public health supply systems in low- and middle-income countries have had difficulty in conducting more rigorous analysis of multiplicity. Data and methodological constraints have prevailed, even for the 3 examples that we cite. But these constraints are changing, pointing to a learning agenda that can be pursued more productively now.

The challenge is to identify a state of *prudent* multiplicity where the costs of additional supply chains (as in Uganda for different sectors or in Bangladesh for different programs) or supply chain segments (as in Chile for procurement) are justified by better risk management, improved supply chain performance, and improved health outcomes. Regarding the universe (or multiplicity) of supply chains in a country as an overarching system provides a constructive framework. Agile use of supply chain resources (for procurement, warehousing, transport, and so forth), wherever they are found and capable to the task, is key to reducing risk and improving performance.

How to find that “sweet spot?” Methods to measure the performance of public health supply systems are well established, and costing methods in the data-poor environments of low- and middle-income countries have recently begun to emerge.^23^-^25^ Likewise, evaluation and modeling of risk in these supply systems is growing.^26^ And methodologies now exist to link supply system performance to health outcomes, at least in the family planning field^27^-^28^—particularly important when considering the economic savings from improved health outcomes enabled by greater availability of commodities to patients, which may outweigh any increased costs associated with multiple supply systems.

Methodological advances now support a more rigorous assessment of the role that multiplicity can play in public health supply systems.

By this confluence, a more structured, holistic approach to assessing, designing, and testing multiplicity is within reach. We suggest 2 avenues:

1. **Retrospective studies** of multiple supply systems may be possible in more data-rich environments.
2. **Intervention research** is also needed to test how multiplicity can be purposely built into public health supply systems to optimize across cost, risk management, and performance. An example of this kind of intervention research is a pilot project in Zambia that evaluated the effectiveness of different approaches for delivering essential medicines.^29^

A greater analytical understanding of the role of multiplicity in public health systems is critical in preparing the systems of the future to handle the growing numbers and volumes of commodities to meet customers' needs.^30^-^31^ As with any significant health system transformation, increased acceptance of multiplicity requires more than just rigorous data and evaluation, and includes advocacy, political support, and leadership to overcome entrenched interests. But the case will best begin with the analytics.

## Footnotes

* Risk management in the supply chain context is the implementation of strategies (of which multiplicity is one) to manage risks along the supply chain (such as a procurement delay, a distributor going out of business, or security threats) based on continuous assessment and with the objective of reducing vulnerability and ensuring continuity of supply to the end customer.^6^
